# Resolving the relative contributions of cistern and pour flushing to toilet water usage: Measurements from urban test sites in India

**DOI:** 10.1016/j.scitotenv.2020.138957

**Published:** 2020-08-15

**Authors:** Claire M. Welling, Siva Varigala, Srinivas Krishnaswamy, Antony Raj, Brendon Lynch, Jeffrey R. Piascik, Brian R. Stoner, Brian T. Hawkins, Meghan Hegarty-Craver, Michael J. Luettgen, Sonia Grego

**Affiliations:** a Center for WaSH-AID, Dept. of Electrical and Computer Engineering, Duke University, Durham, NC 27701, USA; b Dept. Chem. Engineering, BITS Pilani K.K. Birla Goa Campus, Goa, India; c ITC-Kohler Co., Pune, Maharashtra, India; d RTI India, New Delhi 110 092, India; e Biomass Controls, Durham, NC 27701, USA; f RTI International, Research Triangle Park, NC 27709, USA; g Kohler Co., Kohler, WI 53044, USA

**Keywords:** Onsite sanitation, Water reuse, Flow meter, Blackwater, WASH, Decentralized treatment

## Abstract

A challenge in water reuse for toilet flushing in India and other Asian countries derives from pour flushing practices. It is a common assumption that the amount of pour flushed water used for personal cleansing is small in comparison to the cistern flush volume, however there is a knowledge gap regarding the actual contribution of each water source to the blackwater amount. In this study, digital water meters were used to measure the fraction of water from personal wash tap relative to cistern water that is used for toilet flushing. High temporal resolution measurements were carried in three different urban sites in the city of Coimbatore in the southern Indian state of Tamil Nadu where onsite sanitation treatment prototypes that may provide reclaimed water for cistern flushing are being tested. Data collected over periods of up to 2 months show that the contribution of the cistern flush to the total blackwater volume is low (14–40%). These data highlight an important factor to inform interventions designed around water reuse for flushing in world geographies where personal toilet cleansing by water is the common practice.

## Highlights

• The ratio of personal wash vs cistern water used for toilet flushing was measured.
• The pour flush water use averaged 7 L/use.
• The cistern flush contributes to only 14–40% of the volume of water that is flushed.
• Extensive pour flushing will limit the impact of reclaimed water use in cistern flush.

## Graphical abstract

**Figure:**
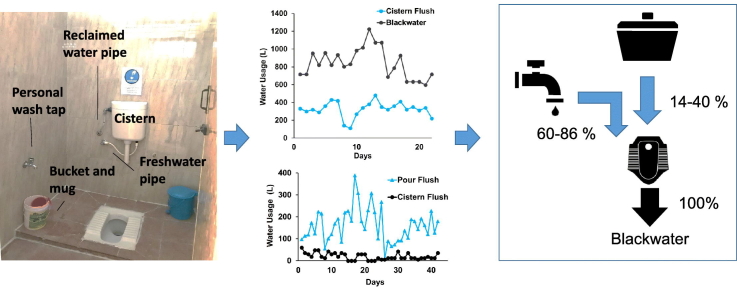

## 1 Introduction

Water treatment and reuse is emerging as a key strategy to address the increased pressure on water resources globally (Opher et al., 2019). However, population growth and accelerated urbanization has led to increased water demand on municipalities while adequate wastewater treatment continues to remain a major global challenge (Voulvoulis, 2018). This problem is of even greater concern in developing countries like India, which is home to 1/7 of the world's population and predicted to face severe water scarcity, possibly starting as early as 2025 (Manna, 2018). In addition, these countries also suffer from lack of access to safe and affordable sanitation.

Greywater recycling offers a potential solution for managing urban wastewater water and can offer savings in the order of 10–50% on water use for residents (Pradhan et al., 2019). Edwin et al. characterized greywater (effluent with no fecal contamination) from several household point sources and assessed its suitability for potential reuse options (Edwin et al., 2014). Their study suggested that treated greywater from bathroom sources alone is enough to fulfill onsite reuse needs and reduce potable water consumption by ~28%. Several studies have also reported on the advantages of greywater recycling for bridging the gap between increasing water supply and demand due to urbanization (Oh et al., 2018; Oteng-Peprah et al., 2018).

Apart from greywater reuse, treated blackwater (the effluent of toilets) is a potential source of non-potable water that can be recycled for toilet flushing and irrigation to address water scarcity. Spearheaded by the “Reinvent the Toilet Initiative” sponsored by the Bill & Melinda Gates Foundation, transformative blackwater treatment technologies have been developed to treat toilet effluent onsite and disinfect the liquid for reuse (Huang et al., 2016; Cid et al., 2018; Rogers et al., 2018; Hawkins et al., 2019; Welling et al., 2020). These emerging technologies aim to produce pathogen-free water for onsite reuse, with the overarching goal of preventing the discharge of untreated wastewater to the environment and India is a major target market.

In India and as well as much of Southeast Asia and other regions, bathrooms are plumbed with a water line for personal cleansing (i.e., toilet paper is not commonly used) (Han et al., 1986; Nawab et al., 2006; Albrecht et al., 2010; Krishnan, 2019). For reuse applications, reclaimed water can only be used for the cistern flush, while the personal cleansing water line must be sourced from fresh water that meets requirements for personal hygiene according to ISO 30500 and EPA guidelines ((EPA, 2012). Thus, in order to appropriately size systems for treating blackwater for reuse, quantitative knowledge of the water used for cistern flushing is needed. The typical amount of water used for cleansing is not well understood, with available data from the literature suggesting a wide range of 0.5-3 L per toilet use (Tilley et al., 2014). Sensor-based monitoring studies (O'Reilly et al., 2015; Zakaria et al., 2018) are rare, and limited to resource constrained environments such as rural latrine (O'Reilly et al., 2015) and emergency camp (Zakaria et al., 2018) .

Cid and co-workers demonstrated toilet effluent treatment prototypes based on electrochemical disinfection technology producing water suitable for reuse as toilet flushing (Cid et al., 2018). A scaled-up version of this technology was designed for an apartment building in India with 20 users and 12 toilet connected to the system. In order to size this onsite blackwater treatment system for the apartment building, coarse assumptions were made: 1. an average number of 5 toilet flushes per person per day (DeOreo et al., 2016) thus this is the maximum number of uses expected in a residential setting; and 2. the 10 L cistern is the dominant factor in toilet effluent volume, and that the combined volume of excreta and cleansing water was approximately 10% of it (~ 1 L).

Based on these assumption the system was thus expected to receive at the most 1100 L/day blackwater. Field measurements over several months revealed blackwater volumes in the range of 700–1400 L/day. However cistern water was estimated to account for less than half of this volume, and no major leaks were identified, thus we hypothesized that pour flushing was extensively practiced even in toilets equipped with cistern flushing.

The objective of this study was to quantify the contribution of cistern flush water to blackwater volume. We additionally intended to elucidate whether bathrooms with squat plates received more pour flushing water than western pedestals, since they are more amenable to receive wastewater from floor cleaning and showering. The study was carried out in the city of Coimbatore in the southern Indian state of Tamil Nadu in India. Data collection took place at three sites where onsite sanitation treatment prototypes that provide treated water for cistern flushing were tested. The installation of these prototypes in residential settings offered the opportunity to connect water flow meters to multiple plumbing lines and record detailed data specific to the water use pattern in the toilets. To the best of our knowledge, this study is the first of its kind and is expected to provide critical input for the design, scale-up, and operation of water re-use.

## 2 Methods

### 2.1 Water use measurement approach

**Fig. 1:**
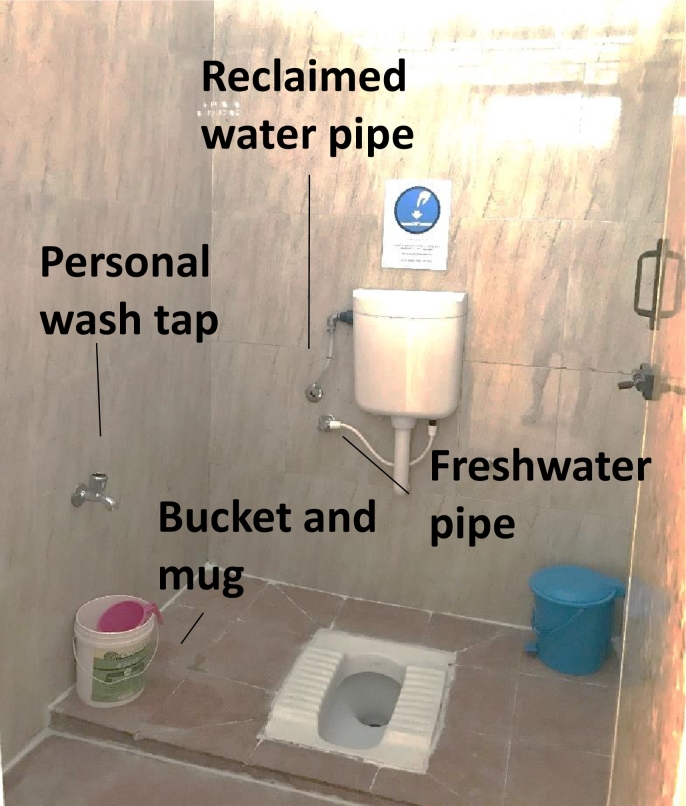
Photograph of an Indian flush toilet used in this study. The stall features an Indian-style squat plate connected to a cistern, the personal wash tap and bucket that can be used for pour flush. A separate water pipe provides reclaimed water to the cistern.

Fig. 1 shows a picture of an Indian flush toilet used in this study with a cistern and personal wash tap and bucket that can be used for pour flushing. Toilets in India are typically equipped with squat pans, although western style pedestal toilets are also available.

Toilet flush water volumes from cistern and from the personal wash tap were measured in connection with the operation of two different treatment technologies in three separate test sites. The Kohler CLASS (Closed Loop Advanced Sanitation System; Kohler Co., Wisconsin, USA) technology was designed to treat blackwater in aggregate from multiple toilets in an apartment building (Varigala et al., 2020). The Duke blackwater treatment system (Center for WaSH-AID, Duke University, North Carolina, USA) was designed to be connected to a single toilet stall for a shared toilet (Welling et al., 2020). A second stall in the same shared toilet facility and not connected to the waste treatment was added to the study as a control for measuring personal water tap use. The procedure for measuring flush water differed between the two technologies due to constraints dictated by the system configuration and access to plumbing lines. Table 1 summarizes the measurement configurations by source and location used in this study, as well as the type of toilet (squat plate or western pedestal) by location.

**Table 1:** Flow meters placements and water measurement configuration by test site.

Site	Location	Toilet	Water to Cistern	Water to Tap	Blackwater
1	Apartment building	12 pedestal	✓	–	✓
2	Apartment building	12 (11 squat plates, 1 pedestal)	✓	–	✓
3	Shared toilet	1 squat plate (test stall)	✓	✓	–
3	Shared toilet	1 squat plate (control stall)	–	✓	–

### 2.2 Measurement system in apartment building

**Fig. 2:**
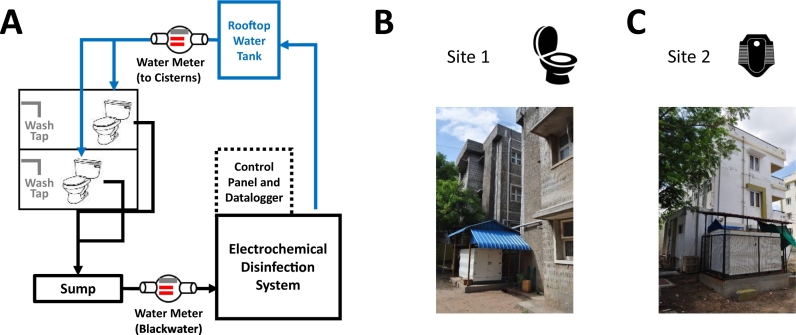
(A) Block diagram of the CLASS onsite blackwater treatment system for apartment buildings; (B) Picture of the apartment complex at Site 1 with adjacent CLASS unit; (C) Picture of Site 2.

The Kohler CLASS was designed to treat toilet effluent from multi-story apartment buildings. The blackwater was collected by gravity and treated by a sequence of biological digestion and electrochemical disinfection (Fig. 2A). Toilet effluent from the building was collected by gravity via a 4″ pipe. In order to measure the volume of effluent, blackwater was collected in a sump and periodically pumped (approximately every 200 L) to the CLASS tanks upon triggering from a level sensor. The treated water from the CLASS was pumped to a rooftop tank. The toilet cisterns drew water by gravity from either of two rooftop tanks: the building water tank or the CLASS treated water tank.

Two CLASS units sized for approximately 1000 L/day blackwater were installed at two residential sites (Fig. 2B and C). Each system was connected to 12 toilets utilized by approximately 20 users. Site 1 was located at PSG Medical College and Hospital campus and housed medical staff. All connected units had western-style pedestal toilets with a 10 L capacity cistern. Site 2 was located on a private senior living facility and housed service staff workers. Apart from one western-style pedestal toilet, all connected toilets were the Indian-style squat plate with a 10 L cistern.

Water measurement were performed in May 2017 at Site 1, while for site 2 water measurements were performed in two separate periods over two years from June 27 to August 4 in 2017 and in October 29 to December 3 in 2018.

The volume of water entering the cisterns was measured by a residential water meter (Zenner MTKD-N25 for 1″ pipe, 10 L resolution) with data logging via a RS-232 serial connection to the control panel of the CLASS. The datalogger wirelessly transmitted readings to a remote data server every 10 min. Custom software was used to access this data server and download the water meter readings and timestamps from which the flow was calculated.

An electromagnetic flow meter (Elmag-06, custom assembled) with flow range up to 5000 L/h and remote digital display was installed along the 1″ pipe from the sump to measure the blackwater entering the CLASS. The flow meter had a PTFE inner lining with SS 316 electrode with remote mount cable transmitter and 4–20 mA output. The flow meter was mounted along a straight section of the pipe, 5 pipe diameters long upstream, and 3 diameter long downstream.

The flow meter control and display unit operated with 220VAC voltage and had a standard communication RS485 port and transmitted the flow rate and total volume in L over time. This data was recorded by the CLASS control panel and wirelessly transmitted to the same server recording the cistern water meter for synchronous recording. The flow meter data was validated and periodically checked and recalibrated against manual flow measurement carried out by pumping a known volume from a container and recording time elapsed.

The flow meter was susceptible to external electromagnetic noises and sensitive to vibrations and the flow meter was re-zeroed by tuning a potentiometer placed inside the transmitter box to attain a steady zero for a no flow case every few months.

### 2.3 Measurement system in the shared toilet

The Duke system was designed to treat onsite effluent from a single toilet. Blackwater was processed by active filtration and electrochemical disinfection. A prototype was installed in a women's toilet block of a private industrial site (i.e., a cotton spinning mill factory). The newly built toilet block was used by female workers, including both day workers and 15–20 workers that resided in the adjacent living quarters. The day workers were local persons from the Coimbatore area, while the resident users moved from other Indian states, mainly Orissa, to be employed. In addition to multiple toilet stalls, the facility included showers and handwashing stations. The toilet stalls featured a squat plate, a personal wash tap, a bucket and mug for personal cleansing, and a 6 L cistern flush (Fig. 1).

**Fig. 3:**
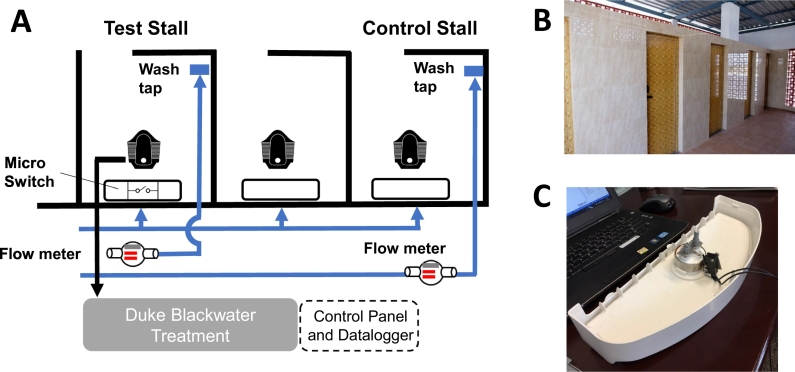
The Duke system is connected to a single toilet within a shared toilet block. (A) Flow meter set-up schematic, (B) External picture of the stalls, (C) Picture of the cistern flush microswitch installed on the inside of the cistern lid.

Fig. 3 shows a diagram of the Duke system installation connected to a single toilet stall within a shared toilet facility. Of the three stalls in use, one (test stall) was connected to the Duke system and the other stalls were plumbed to a septic tank. Two electromagnetic flow meters (Omega, FMG71B-A-BSP) with a flow range of 0.5 to 30 L/min were used to measure water flow in the test stall and one of the other stalls as a control. The flow meters were installed outside of the back wall of the stall, out of sight from the users, to reduce user anxiety that would impact behavior. In the test stall, the flow meter was connected to the 1″ pipe feeding only the personal wash tap (pour flush water). A watertight microswitch (Honeywell,V15W-DZ200A06-AW1) was placed inside the cistern and connected to the flush button. The microswitch was wired through the wall to the treatment control system for the purpose of providing a digital signal once the flush button was depressed to trigger the start of the treatment process. The water use by cistern was obtained by multiplying the flush counts by the volume (6 L) of the cistern flush. A second stall in the toilet block was used as a control and a second flow meter installed along a pipe that fed that personal wash tap (pour flush water). Water use for this study was recorded between July 31 and August 17 2018, and again between October 11 and November 30 2018.

The OMEGA flow meters output a 4–20 mA signal to indicate 0.5–30 L/min flowrate which is read by the controls system and digitized for data collection. Data was sampled every 1–3 s from the flow meters in July–August 2018, but latency caused the data log to miss periods of up to 7 s so the sampling time was increased to 5 s beginning in October 2018. The control panel recorded timestamped flow meter values for both stalls if one of them was non-zero. The local flow meter log was downloaded daily during data collection for this study.

Each flow meter was calibrated with the following procedure. We recorded readings while collecting 1 L of water from the wash tap into a graduated laboratory beaker. To generate data to fit a line, we performed this operation with the tap fully open and half open; each measurement was done in triplicate. The digital values from the flow meter were plotted against the tap flowrates and a linear fit was used to determine calibration coefficients. The calibration was verified before each data collection period, i.e. every 3 months, by repeating the above operation and the drift in coefficient values was found to be <10% (supplementary Fig. S2). Flow meter readings below the 0.5 L/min detection limit of the meter were counted as zero.

### 2.4 Calculation of water use volume for the shared toilet site

A switch indicating the operation of the flush cistern was installed in the test stall in order to trigger operation of the Duke blackwater treatment. This feature enables the controls system to count the number of cistern flushes. The volume of water used for cistern flushing is obtained by multiplying the cistern flush counts by the 6 L volume of the cistern. The effective volume of the cistern was measured to be 6 ± 0.1 L. The cistern water use in the control stall was not measured.

In order to calculate pour flush water use, the instantaneous flow rate (L/min) provided by the Omega flow meter was averaged over the duration of the recorded event. The volume of water generated by this event was calculated by multiplying the event length by the average flow rate. Different thresholds for event separation (10, 15, and 20s) were used, and the resulting volumes differed by <10%. For the subsequent analysis, a separation of 15 s was applied.

### 2.5 Statistical analysis

Data analysis was carried out and plotted in Microsoft Excel 2016. Statistical calculations were performed by GraphPad Prism v8.4 and two-tailed unpaired *t*-tests were used. A *p* value of <0.05 was considered statistically significant.

## 3 Results and discussion

### 3.1 Flush water use in the apartment building sites

**Fig. 4:**
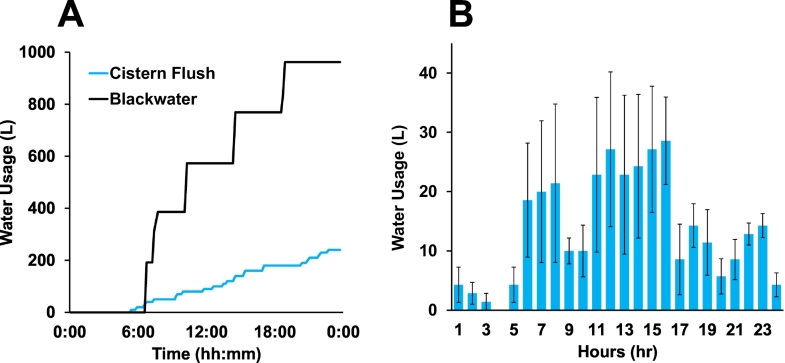
A. A representative 24-h water use record for apartment building site. B. A histogram of water use by cistern flushing averaged over a 7-day period. Errors bars are standard error of the mean.

A representative plot of the water use data collected over a 24-h period in one of the apartment building sites is shown in Fig. 4A. Although water usage was logged every 10 min, the blackwater volume increased in approximately 200 L increments due to the placement of the meter after the sump pump (i.e., a float switch triggered when blackwater was pumped from the sump to the CLASS). The cistern feed was recorded with a resolution of 10 L, which corresponded to the approximate volume of the cistern. A histogram of average hourly cistern use is presented in Fig. 4B. There is little use from 12 AM–6 AM while the residents are sleeping. There is high use from 6 AM-3 PM when the users are awake and preparing from work or returning for lunch. Use is lighter and more distributed throughout the evening.

**Fig. 5:**
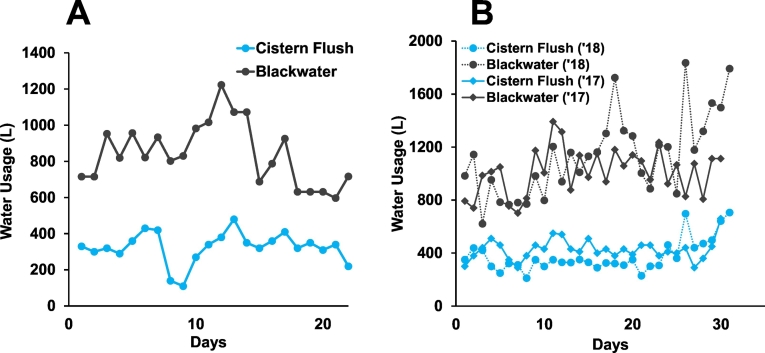
Water use in cistern flushing and blackwater produced in L/day for (A) Site 1 (May 2017) and (B) Site 2 (June–August 2017 and November–December 2018).

To compare cistern water use and blackwater production, total volume was computed over corresponding 24-h time periods. Data was excluded if one of the two logs was missing due to the system being offline or when irregularities, such as pipe breakage, occurred. Collectively, 22 days in May 2017 were analyzed for Site 1 and 61 days were analyzed for Site 2 in June–August 2017 and November–December 2018 (Fig. 5). A two-tailed unpaired *t*-test was used to compare data between 2017 and 2018 for site 2 (Fig. 5b) and the difference was found to be not significant (*p* > .05). The two data sets were combined to calculate the average daily cistern water use and generated blackwater.

Fig. 4, Fig. 5 illustrate that the volume of blackwater produced is consistently much higher than the volume of water used by the cistern flush. At Site 1, the average daily cistern water use was 325 ± 86 L/day, while the amount of blackwater produced averaged 842 ± 171 L/day (*n* = 22). At Site 2, the average daily cistern water use was 401 ± 105 L/day and blackwater volume averaged 1074 ± 257 L/day (*n* = 61).

The difference of 500+ L/day of blackwater accounts for the daily input of urine and pour flush water. According to accepted estimates from literature, the amount of urine produced during one visit to the toilet is 200–400 mL, and most individuals use the toilet five times per day (DeOreo et al., 2016). Assuming conservatively that all 20 users only urinated at home, this accounts at the most for 40 L/day of additional blackwater, which is <10% of the difference in volume between blackwater and cistern flush. This result indicates that the contribution of pour flush water in toilet use is significant and represents over 50% of the source of blackwater. This finding is consistent with the fact that the additional water is used not only for personal cleansing, but also for pour flushing.

### 3.2 Flush water use in the shared toilet site

Water use was analyzed for 13 days in July–August 2018 (data set A) and 42 days in October–November 2018 (data set B), representing 1320 h of monitoring. Only days with a complete 24-h record were included in the analysis (i.e., days were excluded if the log was partially overwritten, power outages occurred, or there were other issues interrupting data recording).

**Fig. 6:**
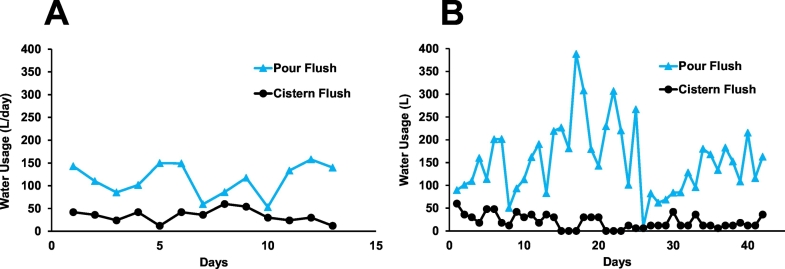
Water use in the shared toilet site by day measured in the test Stall A. Data collected in July–August 2018 and B. in October–November 2018.

While daily water use was found to vary considerably (Fig. 6), the cistern flush water use was consistently lower than the pour flush water volume. The October–November water use overall was increased over the previous period due to increased number of users. A handful of exceptionally long (~30–120 min) use events resulted in peaks consistent with the tap being left partially open after use (Fig. 6b).

**Fig. 7:**
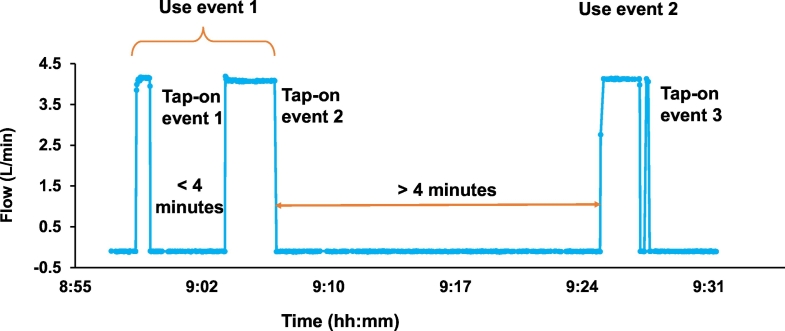
Illustrative examples of flow meter output at Duke shared toilet site 3 versus time of the day. Three Tap-on Events are shown, and two which occur within 4 min of each other are interpreted as one Use Event.

Fig. 7 illustrates a typical flow meter recording over time of day with multiple tap-on events. A user may turn the tap on more than once during a restroom visit, for example to make the squat plate wet before use thereby more easily cleanable, and then multiple times for washing and pour flushing. Literature data suggest the typical residence time of women in a shared toilet stall is 3.8 min (Zakaria et al., 2018). Therefore, a group of tap-on water events within a period of 4 min was chosen to represent the same toilet stall use event. This definition was used to estimate total uses per day. The uncertainty on the total number of uses was obtained as the difference from the same analysis carried out for 3 min and 5 min as the period of stall use. The number of stall visits could in principle be recorded using door switches or pressure sensor on the stall floor. Approaches using electronic sensors located in the stall were deliberately avoided because users' perception of being observed makes them uncomfortable and may impact their behavior.

**Fig. 8:**
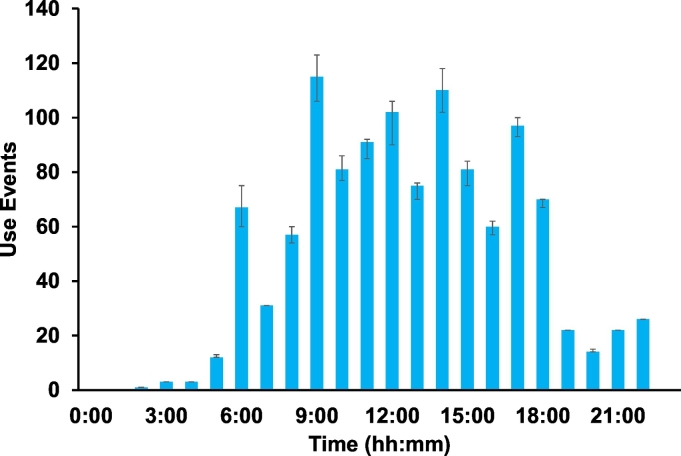
Test stall use events in shared toilet as function of the time of day. Recordings from *n* = 55 days were summed; error bars were obtained as described in text.

The daily pattern of stall visits is shown in Fig. 8. Low use of the stalls was observed from 11:00 PM to 6:00 AM, which is consistent with sleeping patterns. Otherwise, use is distributed during daylight hours as would be expected from a site where users both reside and work.

Table 2 summarizes descriptive statistics from the analysis of all recorded water use data for the shared toilet.

**Table 2:** Water usage at site 3, shared toilet. Values for n = 55 days shown are average ± S.D. Statistical comparisons between the control and test stalls were made by two-tailed unpaired *t*-tests *** = *p* < .001.

Metric	Control	Test Stall		
	Pour flush	Pour flush	Cistern	Pour + Cistern
Water Use (L/day)	262 ± 165	150 ± 70***	25 ± 16	175 ± 67
Flowrate (L/min)	3.8 ± 0.6	2.4 ± 1.0***	–	–
Uses/Day	36 ± 9.5	23 ± 8***	4.2 ± 2.6	23 ± 8
Total Number of Uses	1964	1280^a^	229	1280^a^
Total Water Use (L)	14,419	8230	1374	9604
Water/Use (L)	7.3	6.4		7.5

a These values are the same, since typically cistern flushes occur within the period of one use, thus not representing an additional use.

The average volume of water used per day in the test stall is 175 ± 67 L/Day. The cistern water use represents only 14% of this amount. The use is significantly higher in the control stall than in the test stall in terms of both volume of water consumed and number of uses/day. This difference is likely due to the proximity of the control stall to the entrance of the toilet block (i.e., the control stall is the first one, while the test stall is the third one). The flowrate in the control stall was also significantly higher than the test stall for plumbing reasons, and this may also have contributed to a preference for this stall. Table 2 reports also the recorded number of stall uses and cistern use events. The fraction of recorded cistern flushes during a use event is 229/1280, approximately 18%.

The water used per stall visit was determined by dividing the total water used by the total count of uses. The amount of water used per visit was similar between test and control stall.

The most striking data in Table 2 are the low contribution of cistern flush water to the total water consumed for toilet use at 14% and 18% according to water use and number of event metrics, respectively. An analysis of these values over the two data collection periods revealed that cistern flushing was recorded in 36% of the use events in July–August, but only in 14% of the use events in October–November. We believe that the use of cistern flushing was initially higher because the importance of cistern flushing for the new sanitation system was explained to users in an initial meeting held in March 2018. There was a turnover in users between the two data set collections. Overall, the data indicate a strong preference for pour flushing rather than cistern flushing in this test population.

### 3.3 Comparison of water use across sites

In order to compare the findings across sites, the average daily water contribution of pour flush and the cistern flush for both shared toilet site and apartment buildings is reported in Table 3. The pour flush water use for the apartment building was estimated by subtracting the cistern water use and the volume of human excreta (estimated to be <10% of the total volume) from the total volume of blackwater.

**Table 3:** Water use comparison across 3 Units, n = number of days. Assumes 20 users for each site. ND: not determined. Data are mean ± S.D. Statistical comparisons for all parameters were made by two-tailed unpaired t-tests between Sites 1 and 2 and between the control and test stalls at Site 3.

Site	Location	Total blackwater (L/day)	Pour flush water (L/day)	Cistern water (L/day)	Total water per user (L/person/day)	Cistern fraction of total blackwater
1 (n = 22)	Apartment building,	842 ± 171	495 ± 166	325 ± 86	41 ± 9	40 ± 2%
2 (*n* = 61)	Apartment building	1074 ± 257⁎⁎⁎	652 ± 219⁎⁎	401 ± 105⁎⁎	53 ± 13⁎⁎⁎	38 ± 1%
3 (n = 55)	Shared, Test stall	ND	150 ± 70	25 ± 16	31 ± 13	14%
3 (n = 55)	Shared, Control stall	ND	262 ± 165⁎⁎⁎	ND		ND

⁎⁎ *p* < .01.

⁎⁎⁎ *p* < .001.

All 3 sites had approximately 20 users, although this number was not under our control and it was estimated to oscillate between 12 and 25 during this study. Nonetheless, the figure of 20 users was used to calculate a water/use/day, which is a common metric in the literature and convenient for carrying out comparisons. The calculations for the shared toilet site averaged test and control stall and assumed that the third stall on this site, which was not measured, had a water use equal to the average of the two that were measured.

The water/user/day ranges from 30 to 50 L/user/day and it is comparable across sites and with literature values of 35 L/capita/day for India (Manna, 2018). It should be noted that this measurement includes water use for toilet cleaning, which we are unable to distinguish from regular uses; and the fact that in at least two occasion the tap water was not fully closed after use and kept flowing at low rate overnight.

The fraction of flush water from the cistern is around 40% for both the apartment building sites and it is much lower for the shared toilet (14%). This difference is partially attributed to the larger cistern volume (10 L) in the apartment buildings versus the shared toilet facility (6 L). Another contributing factor may be the fact that the users at the shared toilet facility were mostly migrant workers from other areas of the country where cistern flushing may be less common.

Also, a large variability has been reported in other studies related to toilet use; for example the number of cistern flush per user from different households varied from 3 to 6 flushes/capita/day which was attributed to the diverse age, occupation and customs of the users (Campisano and Modica, 2010).

Total blackwater, pour flush water, and cistern use were all significantly higher at Site 2 than at Site 1. This leads us to estimate that the total water per user per day was also significantly higher at Site 2. Alternatively, there may have been more users at Site 2 than Site 1; we are unable to distinguish between these possibilities based on the data available. However, irrespective of whether these differences are accounted for by different use patterns or different numbers of users, the fraction of the total blackwater attributable to cistern use was not significantly different between these sites. Because the two apartment buildings feature pedestal (site 1) and squat plates (site 2), these data suggest that the type of appliance does not affect the pour flushing volume.

The relative contributions of cistern vs pour flush water on which this study focuses are relatively unexplored, and the literature to provide context to our results is limited. A study from an emergency camp (Zakaria et al., 2018) measured water use from the toilet wash tap as only 0.3 L/use, much lower than the approximately 7 L/use that we recorded; however, the toilet in that study had no cistern and was not in a residential setting with a plumbed water supply. One study of graywater reuse for toilet flushing conducted in a household in Nagpur (India) found that the household produced 55 L/person/day of blackwater and used an average of 25 L/person/day of reclaimed water for toilet flushing, resulting in a cistern water to blackwater ratio of 25/55 = 45% that compares well with the 14–40% we measured (Mandal et al., 2011). However, this study did not address the presence or use of a personal wash tap in the bathroom.

Studies estimate the urban water savings deriving from graywater reuse for toilet flushing between 10 and 30% (Friedler, 2004),(March et al., 2004),(Lazarova et al., 2003). However these studies were conducted in geographies where toilet paper is the common practice (wiping culture) and the cistern is the only source of water for toilet flushing. Our data suggests that in a washing culture, this water savings may be significantly reduced by pour flushing.

## 4 Conclusion

It has been widely proposed that non-potable water reuse for toilet flushing could alleviate pressure on water resources in urban areas. However, our data from three different sites in India found that cistern flushing contributed no >40% of the water flushed, and was as low as 14%. These findings indicate that pour flushing of fresh water was extensively practiced even in bathrooms with cistern toilets installed. Additionally, the type of toilet (squat plate vs western pedestal) did not appear to affect this practice. Thus, low utilization of cistern flushing where pour flushing is practiced may limit the effectiveness of onsite treatment technologies designed to use reclaimed water for toilet flushing as a water conservation strategy, with water savings limited to a small percentage of the total household water demand. This suggests that in washing cultures particularly, alternative water reuse applications such as garden irrigation should be considered and may be preferable.

The large fraction of pour flushing water present in blackwater should also inform the design of onsite blackwater treatment systems for these types of markets. As a direct consequence of this study, the CLASS electrochemical blackwater treatment prototype was redesigned to include automated brine injection because of the dilution of reused water feeding toilet cisterns with pour flush water (Varigala et al., 2020).

While this study was limited in terms of geographic coverage, data were collected over several months and use trends were consistent. This study did not consider any interventions to limit the pour flushing practice, such as providing a spray bidet instead of a tap and bucket. Such interventions may be necessary to improve the impact of household water reuse technologies, particularly in water-scarce regions. This work also suggests that further and more systematic evaluation is needed to fill the knowledge gaps regarding water use for cistern flush by both geography and social context.

## CRediT authorship contribution statement

**Claire M. Welling:** Data curation, Visualization, Writing - original draft. **Siva Varigala:** Methodology, Investigation, Writing - original draft, Writing - review & editing. **Srinivas Krishnaswamy:** Supervision, Writing - review & editing. **Antony Raj:** Investigation, Resources. **Brendon Lynch:** Methodology, Resources. **Jeffrey R. Piascik:** Methodology, Resources. **Brian R. Stoner:** Methodology, Funding acquisition, Supervision, Project administration. **Brian T. Hawkins:** Formal analysis, Writing - review & editing. **Meghan Hegarty-Craver:** Data curation, Formal analysis, Visualization, Writing - original draft. **Michael J. Luettgen:** Methodology, Funding acquisition, Supervision. **Sonia Grego:** Conceptualization, Visualization, Supervision, Writing - original draft, Writing - review & editing.

## Appendix A Supplementary data

S1: Personal wash tap water lines and flow meter placement for the test stall and control stall in shared toilet block. S2: Flow Calibration A. Control Stall, B. Test Stall. Tap water flow measured by recording volume and time are plotted against corresponding digital values recorded from the flow meter. The linear fit equation is used as calibration curve, to convert flow meter recordings into water flow values. Image 1
