# Supplementary material for: Resolving the relative contributions of cistern and pour flushing to toilet water usage: Measurements from urban test sites in India
Source: Sci Total Environ. 2020 Aug 15;730:138957. doi: 10.1016/j.scitotenv.2020.138957 (PMC7272130; doi:10.1016/j.scitotenv.2020.138957)
Supplement: Supplementary file 1 — S1: Personal wash tap water lines and flow meter placement for the test stall and control stall in shared toilet block. S2: Flow Calibration A. Control Stall, B. Test Stall. Tap water flow measured by recording volume and time are plotted against corresponding digital values recorded from the flow meter. The linear fit equation is used as calibration curve, to convert flow meter recordings into water flow values. [file mmc1.docx]

# SUPPLEMENTARY FIGURES


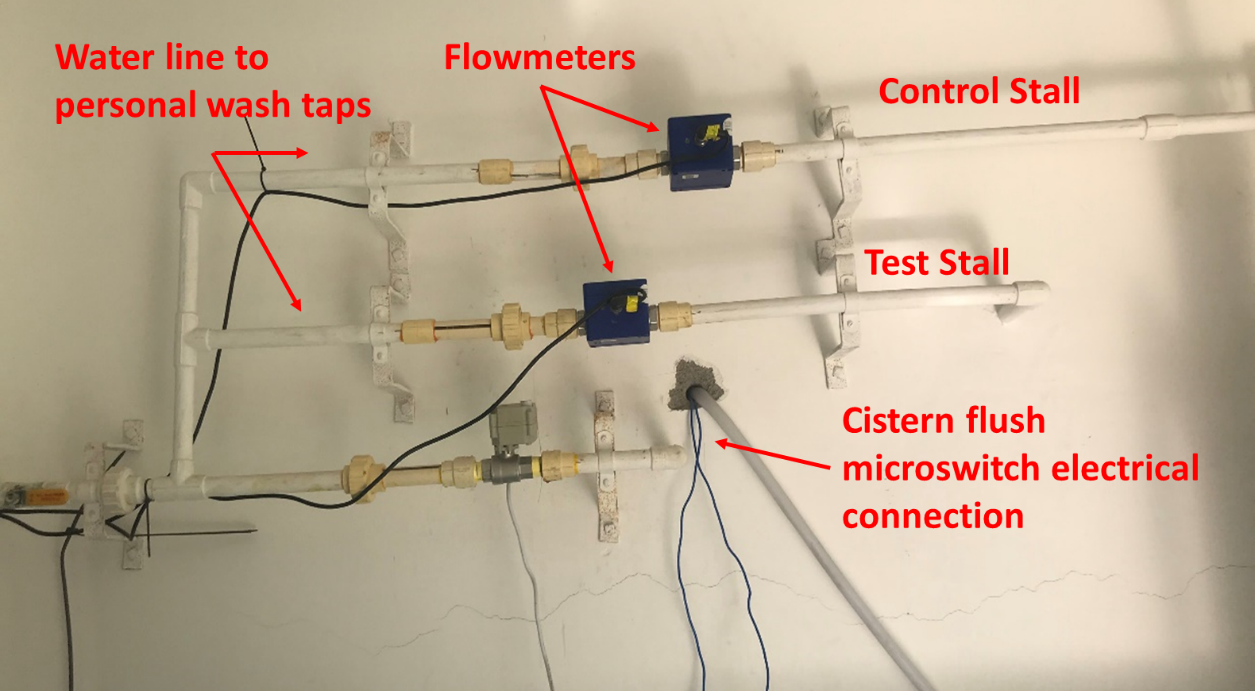


Figure S1 Personal Wash tap water lines and flow meter placement for the test stall and control stall in shared toilet block.


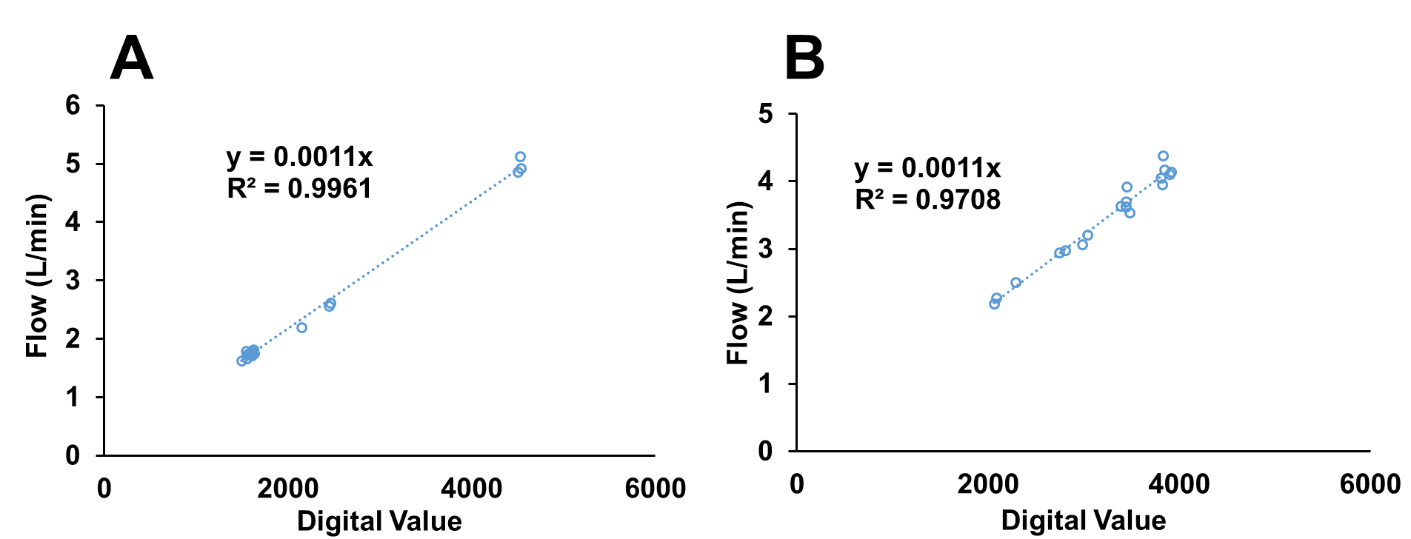


Figure S2 Flow Calibration A. Control Stall, B. Test Stall. Tap water flow measured by recording volume and time are plotted against corresponding digital values recorded from the flow meter. The linear fit equation is used as calibration curve, to convert flow meter recordings into water flow values.
